# Merkel Cell Polyomavirus: Infection, Genome, Transcripts and Its Role in Development of Merkel Cell Carcinoma

**DOI:** 10.3390/cancers15020444

**Published:** 2023-01-10

**Authors:** Roland Houben, Büke Celikdemir, Thibault Kervarrec, David Schrama

**Affiliations:** 1 Department of Dermatology, Venereology und Allergology, University Hospital Würzburg, Josef-Schneider-Straße 2, 97080 Würzburg, Germany; 2 Department of Pathology, Centre Hospitalier Universitaire De Tours, INRA UMR 1282 BIP, 37200 Tours, France

**Keywords:** Merkel cell carcinoma, polyomavirus, T antigen

## Abstract

**Simple Summary:**

By studying the cancer-inducing ability of polyomaviruses, several milestones in cancer research crucially contributing to our current understanding of, e.g., the tumor suppressor proteins p53 and RB1 have been achieved. However, only with the discovery of Merkel cell polyomavirus (MCPyV) and its linkage to the highly aggressive Merkel cell carcinoma (MCC) in 2008 has a human polyomavirus-induced cancer been identified. Since then, intensive research has uncovered many details of the interaction of the virus with its human host, as well as many molecular mechanisms by which the MCPyV-encoded oncoproteins the so-called T antigens mediate oncogenic transformation. Surprisingly, many differences to the previously known polyomaviruses have been observed. In this review, we summarize the current knowledge on MCPyV and MCC and discuss some of the open questions.

**Abstract:**

The best characterized polyomavirus family member, i.e., simian virus 40 (SV40), can cause different tumors in hamsters and can transform murine and human cells in vitro. Hence, the SV40 contamination of millions of polio vaccine doses administered from 1955–1963 raised fears that this may cause increased tumor incidence in the vaccinated population. This is, however, not the case. Indeed, up to now, the only polyomavirus family member known to be the most important cause of a specific human tumor entity is Merkel cell polyomavirus (MCPyV) in Merkel cell carcinoma (MCC). MCC is a highly deadly form of skin cancer for which the cellular origin is still uncertain, and which appears as two clinically very similar but molecularly highly different variants. While approximately 80% of cases are found to be associated with MCPyV the remaining MCCs carry a high mutational load. Here, we present an overview of the multitude of molecular functions described for the MCPyV encoded oncoproteins and non-coding RNAs, present the available MCC mouse models and discuss the increasing evidence that both, virus-negative and -positive MCC constitute epithelial tumors.

## 1. Merkel Cell Polyomavirus (MCPyV) as a Member of the Polyomavirus Family

In the 1950s, substances from cell-free extracts from leukemic mice and released from tissue cultures of mouse tumors could induce multiple neoplasms in mice [1,2]. This substance was believed to be a virus and was later referred to as “SE polyomavirus” [3,4]. Similarly, hamster and raccoon polyomavirus can cause tumors in their natural hosts [5], and this potential to trigger multiple tumors in mice and hamsters led to the name polyomaviridae (“poly” for many and “oma” for tumors) for the respective family. Since these early days, 117 polyomavirus species have been identified, which according to the virus taxonomy 2021 release, are grouped into eight genera (online available at https://ictv.global/taxonomy/ accessed on 9 January 2023). In humans, a total of fifteen family members have been identified [6] and one of them is Merkel cell polyomavirus (MCPyV). MCPyV was discovered via digital subtraction transcriptome analysis in Merkel cell carcinoma (MCC), a tumor that had been suspicious for a viral cause due to its increased incidence in immune-compromised patients [7]. Indeed, MCPyV was found to be present in the majority of MCC. Importantly, the virus was demonstrated to be clonally integrated into the cancer cell genome indicating that integration was an early event during oncogenesis as it must have occurred before the expansion of the tumor cells [7]. Of note, the only other human polyomavirus associated with cancer is BK polyomavirus (BKPyV). Initially identified in the urine of a renal-transplant patient [8], chronic BKPyV infection is recognized as a potential oncogenic factor of urothelial carcinoma developing under immunosuppression [9,10,11]. Since these cases are even rarer, MCPyV-induced MCC is considered the best model to study polyomavirus-driven carcinogenesis in humans.

## 2. MCPyV Is an Omnipresent Virus

MCPyV DNA can be regularly detected on various surfaces always accompanied by the detection of human DNA indicating that it is an omnipresent virus that can be shed from humans [12]. Indeed, several studies demonstrated that up to 80% of participants’ skin swaps were positive for MCPyV, although the results differed on whether there is a prevalence for environmentally exposed and unexposed anatomical sites [13,14,15,16]. Nevertheless, both short- and long-term persistence has been reported for MCPyV in skin swaps, which was associated with elevated viral DNA loads implying that the skin may serve as the major reservoir for MCPyV [16,17]. In addition to normal skin, MCPyV DNA has also been isolated from respiratory, urine, and peripheral blood samples although with a generally lower prevalence compared to skin swaps [18,19,20]. Still, other cells in the body could be a reservoir for MCPyV. In this regard, in a study with two patients, the authors detected MCPyV DNA specifically in inflammatory monocytes, which might allow the spreading of the virus along the migration routes of those inflammatory monocytes [21].

In accordance with the widespread distribution of MCPyV, antibodies against the viral capsid protein VP1 (viral protein 1) are common with a seroprevalence of already 45% in children under the age of 10, which increases throughout life reaching almost up to 90% in adulthood [22,23]. Infections with MCPyV are typical asymptomatic with no signs, symptoms or routine diagnostic test results associated with MCPyV infection [24].

## 3. Infection of Host Cells and Integration in Merkel Cell Carcinoma

**Figure 1:**
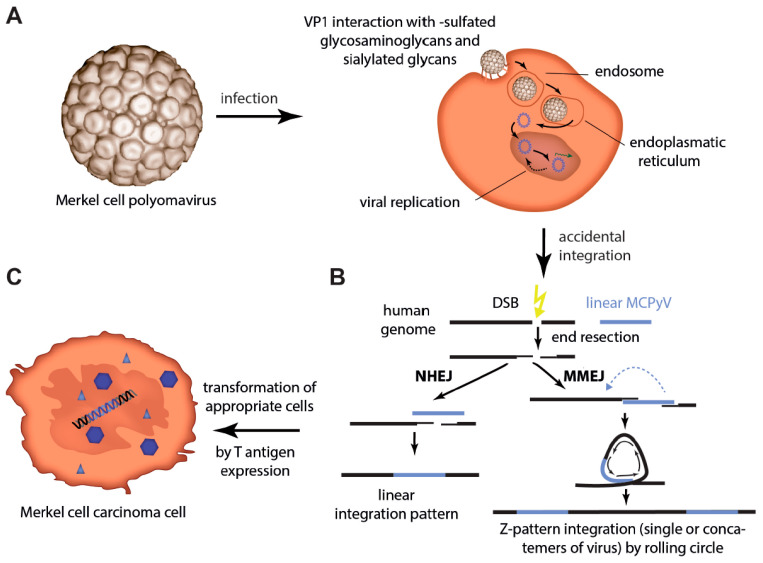
Merkel cell polyomavirus infection and integration. (**A**) After binding of VP1 to sulfated glycosaminoglycans for initial attachment followed by secondary interaction with sialylated glycans, MCPyV enters cells via caveolar/lipid raft-mediated endocytosis. Internalized in small endocytic pits the virus is routed via endosomes to the endoplasmic reticulum probably necessary for uncoating and translocation into the cytosol. Nuclear entry requires mitotic activity of the host cells [28]. (**B**) Integration into the host cell genome is not part of the polyomavirus life cycle. However, in a random genetic accident integration of genomic sequences of MCPyV can occur. Errors during the process of the bidirectional virus replication allow rolling circle amplification or double-strand breaks (DSB) and recombination to cause linear defective viral genomes, which may be present as concatemers. After DSB in the host genome, those linear virus genomes can be ligated into the human genome by either non-homologous end joining (NHEJ) (linear integration pattern) or microhomology-mediated end joining (MMEJ). The latter will result in amplification of host sequence around the integration site (Z-pattern integration) [31,32]. (**C**) When integration of the virus occurs in an MCC progenitor cell, T antigen expression will initiate its transformation.

For infection, MCPyV has to bind through its viral capsid protein VP1 to sulfated glycosaminoglycans for initial attachment followed by secondary interaction with sialylated glycans as entry co-factors [25,26,27] (Figure 1A). Like most polyomaviruses, the internalization of MCPyV is through caveolar/lipid raft-dependent endocytosis, followed by transport in endocytic pits to endosomes from which only a small proportion will get to the endoplasmatic reticulum [28,29]. The ER facilitates capsid uncoating, and the viral genome is then transported via nuclear pore complexes into the nucleus [29]. Subsequently, the proteins of the early region, the T antigens (TA) are expressed. Cellular ubiquitin ligases recognizing conserved phosphorylation sites of Large T antigen (LT) and thereby mediating LT’s degradation can establish viral latency. Upon cellular stresses, the activity of those ligases is reduced allowing LT accumulation to levels that permit the assembly of the replication complex on the viral origin of replication. This accumulation will first initiate virus DNA synthesis and later capsid protein expression followed by cell lysis and release of viral particles [30].

In cell culture experiments, MCPyV-based viral particles can infect primary keratinocytes and a wide variety of transformed cell lines, but the most efficiently transducable cell lines could not support robust replication of MCPyV virions [33]. MCPyV infection is at least in cell culture experiments stimulated by β-catenin and growth factor (e.g., EGF and FGF) signaling inducing matrix metalloproteinase genes [34]. When MCPyV viral particles were applied to a total cell population of the human foreskin, again infection of a wide variety of different cell types including keratinocytes, mesenchymal cells, and fibroblasts was observed. However, replication of MCPyV virions was restricted to dermal cells with dermal fibroblast supporting viral transcription and replication [34]. Whether fibroblasts are, however, cells, which upon infection can give rise to an MCC, is a matter of debate (see Section 5.4).

With respect to the normal MCPyV life cycle it is an accident, but for virus-positive MCC integration of an MCPyV genome encoding a truncated LT is considered as the predominant causal event for cancer evolution and persistence [35,36,37] (Figure 1B,C). Several observations sustain this view: (i) a clonal pattern of the viral genome within the tumor genome [7,38,39,40] indicates that the viral integration occurs before tumor progression and is, therefore, essential for MCC development; (ii) LT in MCC is always truncated but on the other hand its RB1 interaction domain is always preserved indicating that this pattern is essential for MCC development [41]; (iii) in addition to the initiation, growth of MCPyV-positive MCC cells generally depend on MCPyV TAs expression [42,43]; (iv) Transforming ability of MCPyV TAs in vitro and in vivo has established them as oncogenes [42,44,45]; and (v) the lack of recurrent mutations in established human oncogenes in virus-positive MCC [31,46,47] suggests that there might be no crucial genetic contribution to oncogenesis other than MCPyV integration. Although all the given arguments are in favor of the MCPyV TAs being the critical drivers of MCC oncogenesis, testing this hypothesis was limited by the fact that the cell of origin of MCC is still not known (see Section 5.4).

Integration into the host genome is probably a result of errors during the process of the bidirectional virus replication allowing rolling circle amplification or double-strand breaks (DSB) and recombination to cause linear defective viral genomes, which might be present as concatemers. After DSB in the host genome, two different mechanisms of integration of these linear virus genomes have been proposed: ligation to the human genome by non-homologous end joining (NHEJ) will result in a linear integration pattern whereas microhomology-mediated end joining (MMEJ) will result in amplification of host sequence around the integration site resulting in a Z-pattern integration [31,32]. Given that tumors that present with concatemeric viral integrants always contain the same truncating mutation, this mutation has to occur before integration of the viral DNA [39,40].

## 4. Viral Gene Products

**Figure 2:**
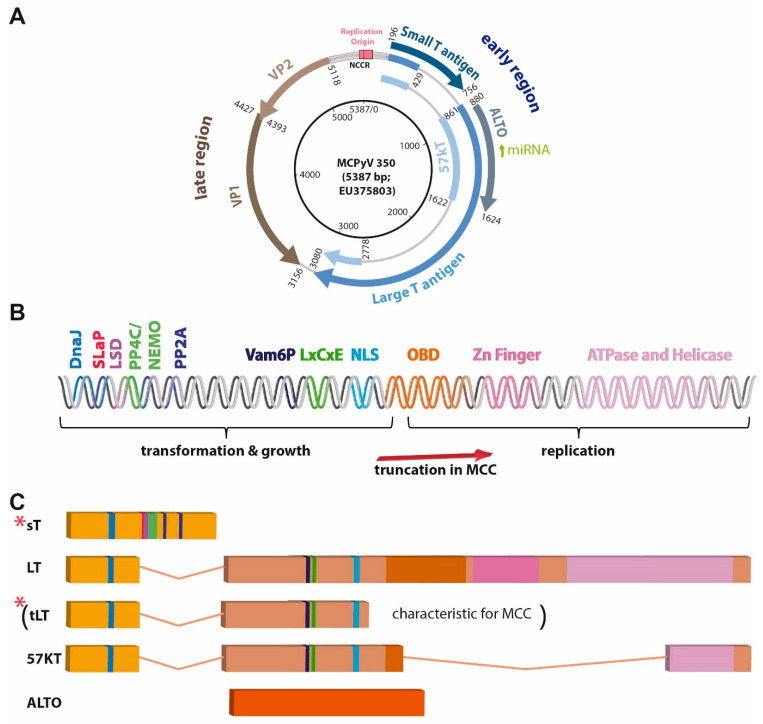
MCPyV genome, described domains, and motifs present in the T antigen region, and T antigen transcripts. (**A**) Map of circular MCPyV. (**B**) Linear map of T antigen (TA) region encoding the depicted motifs and domains. DNAJ: contains HSC70 binding site; LxCxE: RB1 interacting site; LSD: LT stabilizing domain; NLS: nuclear localization signal or sequence; OBD: origin-binding domain; SLaP: ST, MYCL, and P400 complex; PP4C/NEMO, PP2A, Vam6P: respective binding sites, (**C**) differently spliced or open reading frame derived T antigen transcripts. Importantly, due to stop codon mutations or integration-related deletions most often only truncated LT (tLT; specific for virus-positive MCC) and sT (indicated by red stars) are expressed in MCC.

All polyomavirus species contain a single circular double-stranded DNA genome of only approximately 5 kb coding for 5–9 proteins. The genome consists of two distinct transcriptional units located on opposite strands, i.e., the early region encoding the so-called T antigens and the late region encoding the structural viral proteins that form the viral capsid [48] (Figure 2A). Between the early and late regions, the non-coding control region (NCCR) is located, which contains a bidirectional promoter and the origin of replication [49].

### 4.1. Capsid Proteins

Most polyomaviruses express three capsid proteins, which form the viral shell. Of these, VP1 is the major capsid component making up 70% of the total viral protein content, while VP2 and VP3 are required for stable assembly of the capsid [50]. VP3, however, appears to be missing in MCPyV [51] while in some other polyomaviruses a fourth open reading frame can be found in the late region encoding VP4 (Agno protein), which may also be integrated into the viral particle but additionally functions in virus release and induction of apoptosis of the host cell [52].

### 4.2. T Antigens

The T antigens are multifunctional proteins controlling much of the intracellular part of the viral life cycle, which includes (i) regulation of viral and cellular transcription, (ii) viral DNA replication, (iii) virion assembly, (iv) repression of immune responses directed against infected cells and in particular (v) alteration of the cell cycle of the host cell [53] (Figure 2B,C). The importance of the latter, i.e., T antigens’ ability to regulate signaling pathways driving cell cycle progression, can be explained by the dependence of the virus on the DNA synthesis machinery of the host cell for its own replication. In turn, this capability of polyomaviruses to induce cell cycle progression probably explains largely their transforming potential.

Until today, there is only limited information on how the expression of MCPyV T antigens is regulated. In general, NCCR reporter experiments revealed that MCPyV-NCCR-derived reporters showed high activity with regard to early region transcription among the different polyomavirus-derived reporters, and its activity could be further increased by expression of SV40 LT [54,55]. Between MCPyV-NCCR variants, promoter activity varied up to twofold in transfection experiments in dermal fibroblasts. In the same work, the authors demonstrated that while full-length LT did decrease promoter activity, truncated LT was able to increase it [6]. Host factors involved in the regulation of NCCR activity, however, have rarely been reported. In this regard, we could demonstrate that GSK3 inhibition reduces T antigen expression on mRNA and protein levels, although the exact mechanisms are not yet clear [56].

Despite significant variations in the early region sequence between different polyomaviruses, all seem to encode a small T antigen (sT) and a Large T antigen (LT), which are characterized by a common N-terminus and a differentially spliced C-terminus [57] (Figure 2C). However, there is a lot of variation among the different polyomavirus species, with respect to further splice variants derived from the LT-coding sequence (cds) [58] (e.g., 17 kT in SV40 or 57 kT in MCPyV; Figure 2C). A further group of proteins encoded by the early region of some but not all polyomavirus species are partially or completely comprised of a polypeptide derived from an out-of-reading-frame sequence of the second LT exon. The latter is true for alternative T open reading frame (ALTO) of MCPyV [59] (Figure 2C), while Middle T (MT) proteins are derived from an mRNA splice product whose cds consists of most of the sT cds spliced to a second exon equivalent to ALTO [60]. Interestingly, the importance of the different T antigens (at least with respect to their tumorigenic potential) varies among the different polyomaviruses. While MT is the dominant transforming component of murine polyomavirus, SV40-driven transformation is predominantly mediated by LT [61]. In the case of MCPyV, sT seems to bear the strongest transforming potential, as demonstrated by in vitro transformation assays [42] and transgenic mice [44].

For a long time, polyomaviruses, in particular, SV40 and mouse polyomavirus have been used very successfully as model systems to study basic principles of oncogenesis. Indeed, research on SV40 LT contributed crucially to our knowledge of the function of the tumor suppressor RB1 [62], and also p53 was actually discovered through its interaction with SV40 LT [63]. Furthermore, studies on MT have revealed the roles of tyrosine kinases and phosphoinositide 3-kinase (PI3K) signaling in mammalian growth control and transformation [61].

Therefore, when MCPyV was discovered in 2008 research on its gene products could be undertaken on a strong background of knowledge already gathered for other T antigens in particular those from SV40. Interestingly, it turned out, that the MCPyV T antigens are in many aspects different from their SV40 counterparts (see Section 4.2.1 and Section 4.2.2).

#### 4.2.1. Large T Antigen

In SV40-induced tumors, expression of a full-length LT is observed. In contrast, due to point mutations or deletions—a C-terminally truncated MCPyV-LT variant (tLT) is generally expressed in MCC [41] suggesting that its C-terminus bears growth-inhibitory activity [64,65].

As mentioned above, SV40 LT can bind and inactivate p53. MCPyV LT—in particular tLT—seems not to bear such an ability [64,66,67], but in contrast, is activating this tumor suppressor protein [68]. In this respect, the C-terminal helicase-containing region of MCPyV LT has been described to activate the DNA damage response leading to p53 phosphorylation at Ser15 and induction of p53 downstream genes [65]. Furthermore, it has been reported that p53 activation by MCPyV tLT is related to RB1 inactivation, which promotes the upregulation of ARF, a negative regulator of the p53 repressor MDM2 [68]. A last described mechanism of p53 activation by MCPyV LT involves binding to the ubiquitin-specific protease (USP) 7, which has been revealed via pulldown assays [69]. USP7 can normally de-ubiquitinate MDM2 resulting in reduced p53 levels, but tLT binding to USP7 negatively affects MDM2 levels, thereby increasing p53 [69]. Notably, this paragraph only describes the effect of tLT on p53 activity. Later on, the inhibitory effect of sT on p53 activity is presented (see Section 4.2.2). Indeed, TA knockdown in MCC cell lines did not significantly impact p53 reporter activity implying that these two effects seem to level each other out at least in this system [67].

USP7 has also been identified as a negative regulator of MCPyV DNA replication. This does not involve the enzymatic activity as a ubiquitinase, but binding to USP7 increases the affinity of LT to the origin of replication, subsequently limiting DNA replication [69].

**Figure 3:**
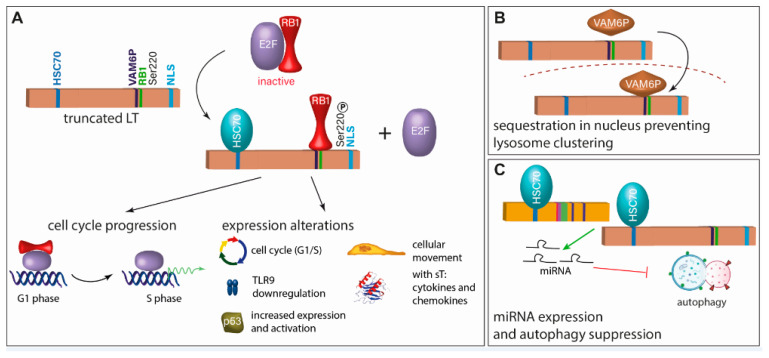
Inhibition of RB1 is the crucial function of truncated LT (tLT). (**A**) For the growth-promoting function of truncated LT, it requires an intact RB1 binding site, has to interact with HSC70, and has to be phosphorylated at Serine 220 [70,71,72]. This leads to cell cycle progression and expression and activation of several molecules. (**B**) Another described function of tLT is binding of VAM6P (VPS39), which leads to its sequestration in the nucleus preventing lysosome clustering [73]. (**C**) tLT and sT induce expression of miRNAs which inhibit autophagy. This function is dependent on the interaction with HSC70 [74].

Several polyomavirus LT proteins such as SV40, JC, and BK are known for binding and inactivating pocket proteins through their LXCXE motif thereby promoting activation of E2F transcription factors and leading to cellular proliferation [62]. Indeed, also the LXCXE motif of MCPyV LT is essential for promoting MCC growth [43] (Figure 3A). However, while SV40 LT can bind and inactivate all three members of the pocket protein family (RB1, p107, and p130), MCPyV LT appears to have a binding preference for RB1, and inactivation of RB1 may be the only essential function of MCPyV LT to support the growth of established MCC cells [70]. Indeed, LT knockdown in MCC cells can be rescued by the RB1 knockdown [70].

Interestingly, MCPyV-LT can specifically bind Vam6p, a factor promoting lysosome clustering and fusion [75], and targets it to the nucleus [73] (Figure 3B). Although the significance of this interaction for viral replication or transformation is not clear, it constitutes a novel function of an LT protein not previously described.

Another difference is that MCPyV-LT contains a nuclear localization sequence (NLS) which is completely different from the prototypic SV40 NLS [76]. This NLS is frequently at least partially lost in the MCC-associated truncated LTs. Nevertheless, probably due to its reduced size, tLT is still able to enter the nucleus and be functional, although instead of sole nuclear localization, both nuclear and cytoplasmic presence are observed [35,72].

The RB1 binding motive in MCPyV LT is flanked by two polypeptide stretches not present in the previously known LT proteins and were, therefore, termed MCV LT unique regions (MURs). It has been proposed that these regions constitute interaction sites for E3 ligases increasing the LT instability [77]. Indeed, comparing full-length LT with one missing the MURs demonstrated an increased half-life of the latter [77].

While viral evasion of autophagy has been described for several human tumor viruses, sT of SV40 has been shown to induce autophagy upon glucose deprivation [78]. For MCPyV, the T antigens induce miRNA expression that targets multiple autophagy genes suppressing autophagy [74] (Figure 3C).

#### 4.2.2. Small T Antigen

**Figure 4:**
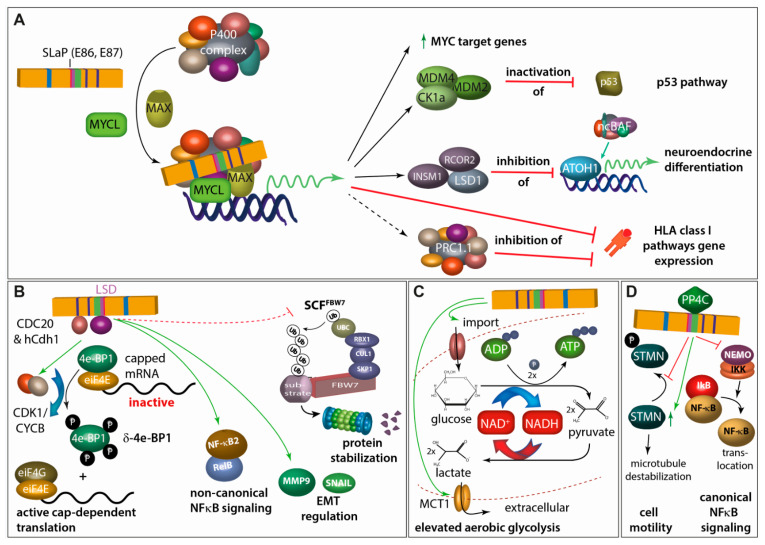
A pleiotropy of functions has been described for sT. (**A**) Via the SLaP binding domain, sT can recruit the transcription factor L-MYC and its heterodimerization partner MAX to the P400 transcriptional regulatory complex. This leads to (i) a general upregulation of MYC-target genes, (ii) inactivation of p53 by increased expression of MDM2 and the MDM4-activator CK1alpha, (iii) expression of LSD1 and other CoREST complex members which in turn repress expression of ATOH-1- and non-canonical BAF (ncBAF) complex-driven expression of genes involved in neuroendocrine differentiation, and (iv) repression of genes involved in HLA class I antigen presentation either directly or through the polyocomb repressive complex 1.1 (PRC1.1) [68,83,84,85]. (**B**) MCPyV sT is a promiscuous E3 Ligase inhibitor. By interacting with cdc20 homolog 1 (Cdh1; hCdh1) E3 ligase adapter and through the LT stabilizing domain (LSD) with CDC20, cyclin-dependent kinase 1/cyclin B1 (CDK1/CYCB1) is activated which leads to hyperphosphorylation of eukaryotic initiation factor 4E (eIF4E)-binding protein (4E-BP1) translating into active cap-dependent translation and increased cell mitogenesis. This effect is contributing to the transforming capacity of sT [42,86]. Moreover, through its LSD domain, sT activates non-canonical NFĸB signaling both by inducing increases in NFĸB2 and RELB transcription and also by promoting NFĸB2 stabilization and activation [81]. In this regard, LSD-mediated interaction of MCPyV sT with different E3 ligases, possibly including FBW7, stabilizes a multitude of different proteins [80,87,88]. For example, this interaction stimulates differential expression of epithelial–mesenchymal transition (EMT)-associated genes such as MMP-9 and Snail [82]. (**C**) sT expression can profoundly impact expression of metabolic pathway genes, especially those involved in glycolysis. Indeed, the expression of two glycose transporters GLUT1 and GLUT3, and the major monocarboxylate transporter for lactate and pyruvate, MCT1, is increased upon sT expression in fibroblasts [89]. (**D**) sT impacts cell motility, partially by interacting with PP4C leading to upregulation of stathmin (STMN)-mediated microtubule destabilization as well as to remodeling of the actin cytoskeleton mediated by dephosphorylation of β1-integrin [90,91,92]. Moreover, PP4C and sT interaction seems to allow repression of the NFκB essential modulator (NEMO), an adaptor protein inhibiting IκB kinase α (IKKα)/IKKβ-mediated IκB phosphorylation and thus limiting NFκB translocation into the nucleus [93].

Similarly, as for MCPyV LT, several novel molecular features have been described for MCPyV sT. While the protein phosphatase 2A (PP2A) inhibiting character of SV40 sT is considered its most important feature [79], it has been reported that transformation by MCPyV sT does not involve PP2A inhibition [42]. Instead, these authors proposed activation of cap-dependent translation through sT-driven hyper-phosphorylation of the translation repressor 4E-BP1 as crucial for MCPyV-sT functioning as an oncogene (Figure 4B). The same domain is also responsible for another novel feature ascribed to MCPyV sT: the inhibition of the protein ubiquitinase FBW7 and other E3 ligases, thereby stabilizing other oncoproteins. Since one of the oncogenes observed to be stabilized was LT, the domain in sT identified to be crucial for this function has been termed LT stabilization domain (LSD) and has been shown to be essential for tumor formation in mice [44] (Figure 4B). Others have confirmed stabilization of LT by sT through its LSD but have raised doubts that this is mediated via FBW7 [80]. Notably, MCPyV sT is the first polyomavirus protein that has been demonstrated to be capable of activating non-canonical NF-κB signaling (Figure 4B). This function has not only been proposed to be essential for MCC cell growth, but also requires the LSD [81]. Another function of sT depending on LSD is its role in the epithelial–mesenchymal transition regulation [82].

One other major function of MCPyV sT is its ability to bind to MYCL, a member of the MYC proto-oncogene family, and recruit it to the EP400 multi-protein complex containing in total 15 proteins including the MYC heterodimer partner MAX [83] (Figure 4A). The domain for this interaction has accordingly been named SLaP (**S**T, MYC**L**, **a**nd **P**400 complex). The sT-induced change in the composition of the EP400 transcription activator complex leads to profound changes in gene expression. A crucial role of this interaction is suggested by the loss of viability of MCC cells upon interfering with MYCL expression and MYL/MAX heterodimer formation and by the loss of transforming potential of an sT variant not able to bind MYCL [83]. Among the proteins induced by the sT/MYCL/EP400 complex is the p53-specific ubiquitinase MDM2 [68], which can initiate p53 proteasomal degradation. Hence, MCPyV sT appears to counterbalance p53 activation induced by MCPyV LT [68]. The EP400 complex generally increases promoter activity. However, there are also key factors indirectly repressed on a transcriptional level by the sT/MYCL/EP400 transcription activator complex. This is for example mediated by induction of the lysine-specific histone demethylase 1A (LSD1/KDM1A), which represses gene expression induced by the lineage transcription factor ATOH1, thereby possibly avoiding terminal differentiation [84]. Further genes indirectly repressed by sT include several class I antigen presentation genes and thus can contribute to immune evasion [85].

Other functions described for sT are the elevation of aerobic glycolysis (Figure 4C), or the increased motility as well as the suppression of the canonical NFkB signaling pathway mediated via the interaction with PP4C (Figure 4D).

Some of this multitude of oncogenic pathways affected by MCPyV sT might explain why it is more potent in the transformation of fibroblasts than SV40 sT [42] and compared to LT is the more potent oncogene of MCPyV [42,44].

#### 4.2.3. ALTO, Circular RNAs and Viral miRNA

The significance of ALTO for MCPyV replication or virus-induced tumor formation remains enigmatic. When viral DNA replication is modeled by transfection of an intact circular MCPyV genome into HEK293 cells ALTO is found to be expressed but appears not to be required for genome replication [59]. In many MCC tumors, the ALTO cds is truncated and therefore, cannot be expressed, and it is unclear whether significant levels of ALTO are present in the remaining MCCs [35]. Interestingly, however, it has been reported that ALTO is also encoded in circular RNAs (circALTO) derived from the early region, which demonstrate increased stability compared to linear RNA and can be found in MCC cells [94]. Translation of circALTO was demonstrated in HEK293 cells and evidence was provided that ALTO protein can regulate certain promoters. Furthermore, the detection of circALTO in exosomes suggested a possible function of modulating transcription in a paracrine fashion [94]. Abere et al. had also described circular RNAs derived from the early region of MCPyV but—in contrast to Yang and colleagues [94]—did not find evidence that these RNAs were translated into a protein [95]. Instead, they suggested that these circular RNAs would act in the regulation of TA expression in concert with an MCPyV-encoded microRNA (miRNA) [95].

miRNAs are small non-coding RNAs that after incorporation into the RNA-induced silencing complex (RISC), can negatively regulate the expression of transcripts with complimentary sequence [96]. Similar to miRNAs that have been reported for other polyomaviruses [96], two miRNAs (miR-M1-5P and miR-M1-3P) derived from a common precursor (miR-M1) are expressed from a sequence in antisense orientation to the MCPyV TAs [97,98,99]. Because of perfect complementarity, the MCPyV miRNAs bear the ability to repress viral gene expression from the early region [97]. Interestingly, miR-M1 has been described to be essential for long-term episomal persistence of MCPyV and, therefore, may be responsible for its ability to produce life-long infections [99]. Although a possible role in MCC is unclear, expression of MCPyV miR-M1-5p has been detected in 50% of virus-positive MCC [98].

While a target mRNA is sliced upon perfect complementarity with a miRNA, imperfect base pairing results in the inhibition of translation and target mRNA decay [100]. Through the latter mechanisms, a single miRNA can regulate the expression of up to 400 genes [101]. Akhbari et al. demonstrated that MCPyV miR-M1 expressed in 293 cells significantly represses the expression of more than 70 cellular genes, among which they observed several implicated in immune evasion (e.g., the innate immunity protein S100) [102]. Therefore, miR-M1 might contribute to the immune escape of MCPyV-infected cells.

## 5. Merkel Cell Carcinoma

MCC is a rare and very aggressive cutaneous neuroendocrine skin cancer with a greater than 30% 5-year overall mortality rate [103,104]. In accordance, a high rate of metastasis mostly to the lymph nodes or distant organs is one of the well-known features of MCC [103]. This tumor grows in the majority of cases in the dermal layer of the skin. Fair-skinned, elderly, immunocompromised, or people with a history of other cutaneous tumors carry an increased risk of developing this malignancy.

MCC was first described in 1972 by Cyril Toker as “trabecular carcinoma of the skin” [105]. Its current name has been given to the malignancy after recognition of its neuroendocrine features, which render the tumor cells highly similar to epidermal Merkel cells [105]. Indeed, these similarities between Merkel cell carcinoma cells and Merkel cells led soon to the suggestion that the latter might be the origin of the newly discovered tumor [105,106].

### 5.1. Merkel Cells

Merkel cells (MC) are highly specialized skin cells, able to transform mechanic triggers into Ca^2+^ action potentials [107,108] thereby functioning as sensory receptors for light touch stimuli [109]. Located in the basal layer of the epithelium, particularly in areas of highly sensitive skin, MCs are found close to nerve endings, either dispersed around hair follicles or in so-called touch domes, innervated structures consisting mainly of MCs and specialized keratinocytes [110]. A protein expressed by MCs and crucial for their function is Piezo2 serving as a mechanically activatable cation channel [111]. Additionally, MCs are characterized by the expression of neuroendocrine markers such as CD56, synaptophysin, chromogranin A, and INSM1 and distinct epithelial markers such as KRT8, KRT18, and KRT20. [109,112,113]. Furthermore, MCs are the only cells in the skin expressing the transcription factor ATOH1 [114], which has been shown to be crucial for MC development [107]. Indeed, mice with epidermal *Atoh1* knockout driven by Cre recombinase controlled by the *KRT14* promoter resulted in a loss of MC in all regions of the skin [115]. On the contrary, the same setup under the neural crest-specific WNT1 promoter did not affect the growth and development of MCs in mice [115]. Therefore, these experiments not only confirmed the essential role of Atoh1, but also indicated that the cellular origin of MCs is actually an epidermal skin precursor, and not as hitherto debated a neural crest-derived cell. These findings also influenced the speculations on the origin of Merkel cell carcinoma.

### 5.2. MCC: Two Different Tumor Entities

Another twist in the discussion on MCC oncogenesis came from findings suggesting that MCC constitutes actually two different tumor entities [116]. In this respect, already the initial discovery that a previously undescribed polyomavirus was present in eight out of ten MCC patient samples, implicated that MCPyV might be involved in many, but not all MCC cases [7]. Indeed, a recent meta-analysis including 35 publications confirmed an overall pooled prevalence rate of MCPyV in MCC of 80% (95% CI = 71–88%), leaving 20% of cases as virus-negative MCC [117]. Importantly, detailed genetic analysis of virus-positive and -negative MCCs revealed significant differences with respect to mutational load and genome stability [31,46,47]. For example, Goh et al. reported that while MCPyV-negative MCC is among the cancers with the highest mutational burden (median: 1121 somatic single nucleotide variants (SSNVs) per tumor exome), MCPyV-positive MCCs subsume on the other side of the spectrum, with typically very low numbers of mutations (median: of 12.5 SSNVs per exome) [47]. Moreover, since only virus-negative MCCs display typical UV mutational signatures (predominance of C > T exchanges at dipyrimidines) [31,46,47] it has been concluded that MCC derives either via a UV-dependent or via a virus-dependent tumorigenesis pathway. Some authors concluded from the blatant differences regarding the presence and absence of UV exposure-induced genomic alterations that—despite the similarities with respect to phenotype and clinical behavior—only virus-negative MCC can have an epidermal origin [118]. Indeed, others suggested the usage of a novel nomenclature for virus-negative and -positive MCC: Merkel type sarcoma and squamous cell carcinoma, Merkel type, respectively [116]. Another recently reported feature distinguishing the two MCC subtypes might be the differential expression of mismatch repair proteins [119].

### 5.3. MCPyV-Positive MCC: A Virus-Induced Tumor

Although further genetic and epigenetic alterations may contribute to the development of virus-positive MCC, integration of an MCPyV genome encoding a truncated LT is considered the predominant causal event for cancer evolution and persistence [35,36,37] (Figure 2B,C). As discussed before, this view is sustained by (i) the mono-clonal integration of the viral genome within the tumor genome [7,38,39,40], (ii) the preservation of the RB1 interaction domain in the truncated LT [41], (iii) the transforming ability of the MCPyV TAs in vitro and in vivo [42,44,45], (iv) the dependency of established MCC cells on TA expression [42,43] and (v) the lack of recurrent mutations in established human oncogenes in virus-positive MCC [31,46,47] suggesting that there might be no crucial genetic contribution to oncogenesis other than MCPyV integration. Although all the given arguments are in favor of the MCPyV TAs being the critical drivers of MCC oncogenesis, testing this hypothesis was limited by the fact that the cell of origin of MCC is still not known.

### 5.4. The Cellular Origin of MCC

Due to shared neuroendocrine features and immunohistochemical characteristics, it has been initially conjectured that Merkel cells might be the cell of origin of MCC [120]. However, given that Merkel cells are post-mitotic cells, MCPyV integration and transformation, required for viral MCC development and growth, cannot be properly fulfilled. Indeed, it has been shown that MCPyV sT overexpression failed to stimulate proliferation and tumorigenesis in mature Merkel cells [121]. Furthermore, MCCs express markers such as CD171, CD24, and C-kit, which are absent in Merkel cells. Moreover, Merkel cells are found in the epidermis, whereas MCCs are almost always found at the dermal or subcuticular level of the skin [122,123]. Evaluating all these facts, the argument that Merkel cells may be the ancestor cell of MCC has been refuted, and instead, other hypotheses such as epidermal stem cells, dermal stem cells, fibroblasts, and pre-/pro-B cells have attracted attention [124]. In addition, the differences between viral and non-viral MCC in the frequency of UV mutational burdens (as detailed above) have also raised the possibility that the two subtypes may have distinct cellular origin [118]. In conclusion, the question of what the cell of origin of MCC is has become recently a highly controversial topic in the field.

Interestingly, MCC tumors commonly express essential B cell markers such as PAX5, TdT, and C-Kit, suggesting that MCC might be derived from pro-/pre-B cells [125]. In addition, the possible origin of MCCs from B-cells might explain the dermal or even subcuticular localization of MCC, since in contrast to Merkel cells or epidermal stem cells they are not located in the epidermis [125,126]. Moreover, reports of immunoglobulin expression and rearrangement in viral MCCs have also positively contributed to this hypothesis [126].

Another hypothesis proposed by Sunshine and colleagues is that fibroblasts are the cellular origin of MCPyV-positive MCC [118]. Their major arguments were that (i) fibroblasts are the cellular compartment in the skin supporting productive MCPyV infection [34], (ii) the mutation frequency of virus-positive MCC matches that of dermal fibroblasts [118], and (iii) the failure of several mouse models with epidermal targeting of MCPyV TA expression to convincingly recapitulate human MCC [45,121,127].

The authors favoring fibroblasts as the potential origin of MCPyV-positive MCC nevertheless postulate that virus-negative MCC arises from epithelial cells [116,118]. This notion is supported by several recent reports that virus-negative MCC can arise from epithelial tumors [128,129,130]. Indeed, MCPyV-negative MCC is frequently found in close association with squamous cell carcinoma (SCC), either in situ or invasive [131]. Sequencing both compartments of several of such combined tumors identified many mutations shared between MCC and SCC parts, providing compelling evidence that one arises from the other [128,129,130]. Although a genetic event driving this transformation could not be identified, all three reports provide evidence that RB1 inactivation may be a prerequisite, since it is much more frequent in the SCC part of combined tumors than in pure SCC [128,129,130]. Given that a considerable proportion of MCPyV-negative MCC is diagnosed as a combined tumor with an SCC component [131,132], these results strongly suggest that MCPyV-negative MCC is generally a keratinocytic tumor.

Importantly, MCPyV-positive MCC might also constitute a keratinocytic tumor as suggested by comprehensive analysis of DNA-methylation patterns which grouped virus-negative as well as virus-positive MCC cell lines along with epithelial cancers [133]. A keratinocytic origin, despite largely lacking an epidermis-characteristic profile, could be explained by assuming that MCPyV-positive MCC originates from cells protected from the damaging effects of sunlight. Such a population might be hair follicle stem cells or Merkel cell progenitors located in hair follicles, deeply extending into the skin. Indeed, transgenic mice models presented tumorigenic potential in the GLI-positive Merkel cell progenitors, which additionally express KRT17 and SOX9 markers during Merkel cell differentiation in hairy mice skin [134]. A tumor with hair germ differentiation, displaying a significant phenotypical overlap with Merkel cell precursors from the hair follicle is trichoblastoma [135]. Genetic analysis of a very rare, combined tumor consisting of trichoblastoma and viral MCC revealed six common somatic mutations while MCPyV was only detected in the MCC compartment. This observation indicates that the MCC arose upon integration of the MCPyV genome into the genome of an epithelial cell of the trichoblastoma compartment [135]. Since the induction of a neuroendocrine phenotype in epithelial precursor cells had previously been observed when expression of SV40 TAs was targeted to the gut epithelium [136] it appears possible that also MCPyV TAs are capable of mediating such a transdifferentiation.

Further support for Merkel cell progenitors of the hair follicle being the origin of MCPyV-positive MCC comes from a recent MCC mouse model.

### 5.5. MCC Mouse Models

**Figure 5:**
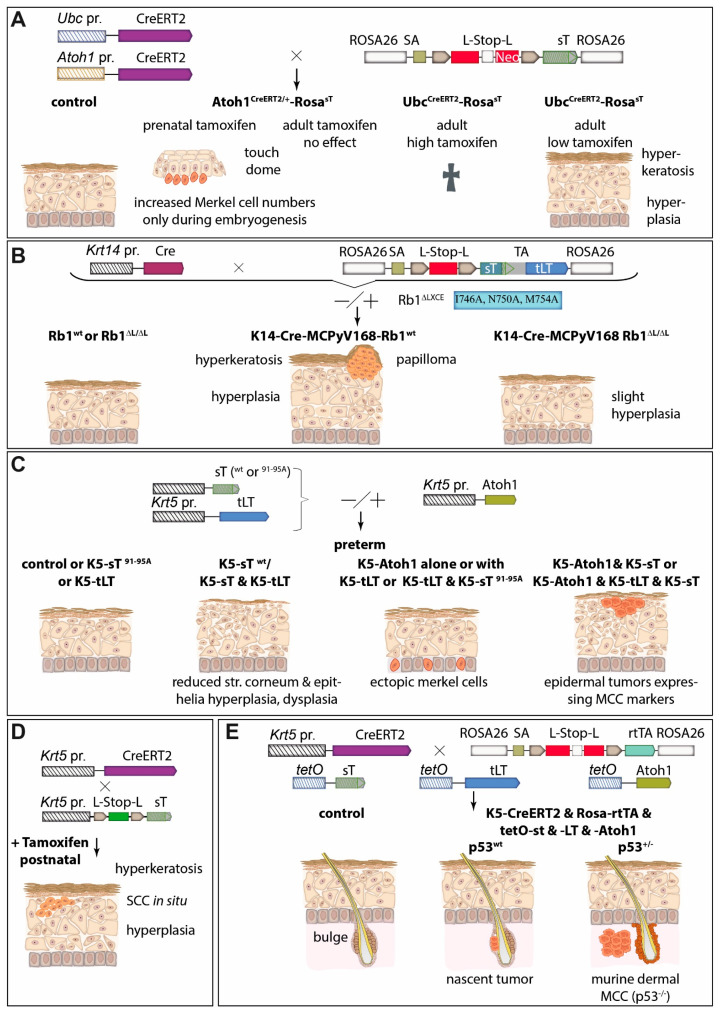
Of the different strategies tested, so far only the combined expression of T antigens and the transcription factor Atoh1 in a p53-deletion background can trigger MCC formation in a mouse model. Several groups have tested the transforming capacity of especially sT in different mouse models. (**A**) Transgenic mice that conditionally express MCPyV sT from the *ROSA26* locus by expressing Tamoxifen-activatable Cre recombinase either ubiquitously (under the *UBC* promoter) or specifically under the promoter of *Atoh1* encoding the master regulator of Merkel cell development. Outcome in these models depends on time point and dose of tamoxifen administration, resulting in either a temporary increase in Merkel cells, death, or epidermal hyperplasia and hyperkeratosis [121]. (**B**) Similarly, using the Krt14 promotor to express MCPyV T antigens in stratified squamous epithelial cells and Merkel cells of the skin epidermis, causes hyperkeratosis and hyperplasia, but in half of the cases, additionally papilloma. Notably, this phenotype is almost completely prevented in an Rb1^ΔLXLC^ background attenuating LT-Rb interactions through LT’s LXCXE motif [45,138]. (**C**) In preterm models, *Krt5* promoter-driven epidermis-targeted sT expression only caused a phenotype with an intact LT stabilization domain and in combination with Atoh1 expression triggered epidermal tumors displaying MCC markers. Interestingly, additional epidermal expression of truncated LT had no impact on the phenotypes caused by Atoh1 and/or sT [44,127]. (**D**) Tamoxifen-induced expression of sT in adult mice drives rapid epidermal hyperplasia and development of skin lesions resembling squamous cell carcinoma (SCC) in situ [127]. (**E**) Conditional expression of T antigens and Atoh1 in epidermal cells initiate nascent MCC-like tumors at hair follicles and dermal MCC when tumor cells lose p53 expression [140]. Abbreviations: Krt or K: keratin; L-Stop-L: loxP-Stop-LoxP; pr.: promoter; tLT: truncated LT; rtTA: reverse tetracycline-controlled transactivator.

Cancer mouse models provide an essential contribution to understanding a specific tumor type by modeling the complex interactions between tumor cells and their host environment. Moreover, they can be used to test new therapeutic approaches. Therefore, several groups tried to recapitulate MCPyV-driven MCC development in mice (Figure 5). (i) Shuda and colleagues demonstrated that ubiquitous MCPyV sT expression plus conditional homozygous p53 deletion led to poorly differentiated tumors in the spleen and liver, while sT and p53 deletion targeted to Merkel cells by utilizing the *Atoh1* promoter did not result in tumor formation [121] (Figure 5A). Two other groups developed their MCC mice under the assumption that epidermal cells should be the cellular origin of MCC by utilizing the promoters of keratin (*Krt*)*5* and *Krt14*, respectively. These two genes are expressed in the basal cell layer of all epithelia [137]. (ii) Using the *Krt5* promoter Verhaegen and colleagues observed sT-induced hyperplasia-lacking expression of MCC markers—in the epidermis and other epithelia in preterm embryos and this was dependent on a functional LSD but not on PP2A binding [127]. In addition, postnatal induction of MCPyV sT in Krt5-positive cells, using a tamoxifen-responsive Cre/Lox system, induced tumors resembling squamous cell carcinoma in situ [127]. In a follow-up paper the same author’s co-expressed sT with the Merkel cell determining transcription factor Atoh1 in developing epithelia and observed a somewhat more MCC-like phenotype of the induced tumors, which, however, unlike human MCC displayed epidermal localization [44] (Figure 5C,D). Importantly, neither Krt5-driven expression of MCPyV LT nor co-expression of MCPyV-LT with Atoh1 or with sT and Atoh1 did alter the phenotype of the mice—in particular tumor growth in the Atoh1/sT setting—questioning the significance of LT with respect to tumor formation [44]. (iii) In contrast, Spurgeon et al. demonstrated in their mouse model that RB inactivation is required for MCPyV TA-induced tumorigenicity, suggesting that LT is playing a major role [138]. They used *Krt14* promoter-controlled Cre expression to produce a functional sT/LT expression cassette in epithelial cells and observed hyperplasia, hyperkeratosis, and acanthosis of the skin, as well as the formation of benign epithelial tumors, named papilloma [45] (Figure 5B). They proposed that the MCPyV TAs function as tumor promoters since they observed synergy in epithelial tumor formation with the chemical tumor initiator DMBA (7,12-dimethylbenz(a)anthracene), but not with the tumor-promoting agent TPA (12-O-Tetradecanoylphorbol-13-acetate) [139]. They conclude from these results that in the human setting other molecular events might be necessary to initiate MCC oncogenesis [139]. Finally, they describe in a recent paper that MCPyV-TA-induced tumorigenesis is abolished when experiments are performed in mice expressing an RB1 with much reduced MCPyV LT binding capability demonstrating for the first time the role of LT in tumor formation [138].

The mouse models described so far have demonstrated the tumorigenic potential of the MCPyV TAs, with contradicting results with respect to MCPyV LT. Moreover, they failed to produce a tumor phenotype resembling human MCC. However, in a recent breakthrough paper, Verhaegen and colleagues now report the generation of mice that develop tumors closely resembling human MCC [140]. To this end, they established adult mice in which expression of sT, truncated LT, and Atoh1 could be turned on in Krt5-expressing cells and their descendants through the administration of doxycycline. Interestingly, despite *Krt5* promoter activity in the basal layer of the complete epidermis, cellular aggregates displaying an MCC-like phenotype were only observed in the hair follicle, close to the stem cell compartment called the bulge [140]. Since these nascent MCCs presented accumulated p53, the mice were next crossed with animals carrying one floxed p53 allele, resulting in mice that developed macroscopic tumors. These tumors had lost all p53, and upon histological examination displayed expression of many human MCC markers and particularly the characteristic dot-like Krt20 staining [140]. Importantly, the tumors, like typical human MCC, were localized within the dermal compartment of the skin without an obvious connection to the epidermis or hair follicles (Figure 5E). In summary, Verhaegen and colleagues have achieved the establishment of a convincing murine MCC model, although it is a pity that they did not address the question of whether MCPyV LT is required in this setting. The necessity of inactivating p53 on a genomic level is different in the human setting where p53 inactivation is supposed to occur via MCPyV sT mediated upregulation of MDM2 [68]. Of specific interest is the finding that the development of MCPyV TA-induced MCC precursor lesions is restricted to the hair follicle niche, which brings strong support to the hypothesis that hair follicle stem cells are the cells of origin of human MCPyV-positive MCC [135] (see Section 5.4).

## 6. Conclusions

Recent years have seen an enormous gain in our knowledge of MCPyV-induced MCC. A multitude of crucial oncogenic pathways targeted by the T antigens has been revealed, and the evidence that virus-positive MCC is an epithelial tumor originating from hair follicle cells is increasing. Certainly, there is still a lack of understanding of which host factors drive T antigen expression. This knowledge is especially desirable since it could translate into new therapies for virus-positive MCCs.
